# Cytokine Immunopathogenesis of Enterovirus 71 Brain Stem Encephalitis

**DOI:** 10.1155/2012/876241

**Published:** 2012-08-23

**Authors:** Shih-Min Wang, Huan-Yao Lei, Ching-Chuan Liu

**Affiliations:** ^1^Department of Emergency Medicine, National Cheng Kung University Hospital, College of Medicine, National Cheng Kung University, Tainan 70428, Taiwan; ^2^Center of Infectious Disease and Signaling Research, National Cheng Kung University, Tainan 70428, Taiwan; ^3^Department of Microbiology & Immunology, College of Medicine, National Cheng Kung University, Tainan 70428, Taiwan; ^4^Department of Pediatrics, National Cheng Kung University Hospital, College of Medicine, National Cheng Kung University, Tainan 70428, Taiwan

## Abstract

Enterovirus 71 (EV71) is one of the most important causes of herpangina and hand, foot, and mouth disease. It can also cause severe complications of the central nervous system (CNS). Brain stem encephalitis with pulmonary edema is the severe complication that can lead to death. EV71 replicates in leukocytes, endothelial cells, and dendritic cells resulting in the production of immune and inflammatory mediators that shape innate and acquired immune responses and the complications of disease. Cytokines, as a part of innate immunity, favor the development of antiviral and Th1 immune responses. Cytokines and chemokines play an important role in the pathogenesis EV71 brain stem encephalitis. Both the CNS and the systemic inflammatory responses to infection play important, but distinctly different, roles in the pathogenesis of EV71 pulmonary edema. Administration of intravenous immunoglobulin and milrinone, a phosphodiesterase inhibitor, has been shown to modulate inflammation, to reduce sympathetic overactivity, and to improve survival in patients with EV71 autonomic nervous system dysregulation and pulmonary edema.

## 1. Introduction

Humoral mediators including cytokines are the molecular proteins of the innate and immune response and play key roles in the pathophysiology of viral infection [1]. Systemic inflammatory response syndrome (SIRS) caused by infection is a typical condition within which proinflammatory mediators released from infected cells and persistent hypercytokinemia may result in progression to multiple organ failure [2]. It is known that activation of cytokine networks increases levels of various cytokines in blood. The burst of cytokine release that follows sepsis, toxin-mediated shock syndrome (e.g., *Streptococcus pyogenes* and *Staphylococcus aureus*) [3, 4], some virus infections such as severe acute respiratory syndrome (SARS) [5], influenza [6], dengue virus [7], and Epstein-Barre virus [8] induce an overwhelming stimulation of innate and/or immune responses that storm the physiology of the body.

## 2. Clinical Manifestation of EV71 Brain Stem Encephalitis

Human enterovirus (EV71) is member of the genus *Enterovirus, *family *Picornaviridae*, which consists of a nonenveloped capsid surrounding a core of single-stranded, positive-polarity RNA approximately 7.5 kb in size and 27–30 nm in diameter [9, 10]. EV71 produces a broad spectrum of clinical manifestations. The majority of infected individuals have asymptomatic infection. Mild cases are characterized as cutaneous diseases such as hand, foot, and mouth disease (HFMD) and herpangina. However, potentially life-threatening neurological complications such as brain stem encephalitis (BE) are of the greatest clinical and public concern [11–14]. EV71 has been recognized as highly neurotropic and associated with a diverse range of neurological diseases, such as aseptic meningitis, BE, encephalomyelitis, acute flaccid paralysis (AFP), and postinfectious neurological syndromes. During the 1998 Taiwan epidemic several clinical stage categories of disease were developed for the severity of BE to help monitor the clinical course of EV71 infection and to aid management. These systems are not, however, widely used, possibly because they are not always easy to follow by primary care physicians. In 2010, World Health Organization Regional Office (WHO) for the Western Pacific and the Regional Emerging Diseases Intervention (REDI) Centre documented guide for clinical management on hand, foot, and mouth disease that has proposed simple clinical stages of disease manifestation to describe the disease severity as we suggested previously [15, 16]. The EV71 BE was stratified into three important critical stages by disease severity, including uncomplicated BE, autonomic nervous system (ANS) dysregulation, and pulmonary edema (PE). It is a continuous and dynamic disease sequence. It may be a reversible disease because each critical stage is a turning point. Through this staging, the pathogenesis of BE was explored and then effective ways to manage the patients were developed. BE is defined as an illness characterized by myoclonus, ataxia, nystagmus, oculomotor palsies, and bulbar palsy in various combinations, with or without neuroimaging. ANS dysregulation is defined by the presence of cold sweating, mottled skin, tachycardia, tachypnea, and hypertension. PE is defined as respiratory distress with tachycardia, tachypnea, rales, and frothy sputum that developed after ANS dysregulation, together with a chest radiograph that showed bilateral pulmonary infiltrates without cardiomegaly. If the diagnosis of EV71 BE once was delayed, usually because of the clinical symptoms are not recognized in the early stages. Myoclonic jerks are seen more often in EV71 than in other serotypes of enteroviruses and could be an early indicator of brain stem involvement. Diagnostic workup of EV71 BE should include the search for one or more neurological symptoms, especially myoclonus jerk and limb paralysis, and the measurement of disease markers, such as peripheral white blood cell count, platelet count, glucose level, inflammatory cytokines, immune cell subsets, and cerebrospinal fluid analysis [17–22]. In the 2008 outbreak of Taiwan, 238 virologically and clinically confirmed severe cases were identified, including 41% uncomplicated BE, 44% ANS dysregulation, and 15% PE [23].

## 3. Pathogenesis of Complicated EV71 Brain Stem Encephalitis

Both innate and adaptive immune mechanisms are important for host defense against viral infection. The innate immune system provides the first line of defense against virus through activation of adaptive immunity through antigen presentation as well as secretion of proinflammatory cytokines. The pathogenesis of PE and hemorrhage in EV71 infections has been studied, with some important findings. Destruction of vasomotor and respiratory centers in the medial, ventral, and caudal medulla by EV71 leads to ANS dysregulation and PE. It is similar to that observed in bulbar poliomyelitis and produces sympathetic hyperactivity, with surge catecholamine and autonomic dysfunction [19, 24]. Catecholamines are among the neurotransmitters that affect immune responses humorally through circulating epinephrine, as well as locally through neuronal release of norepinephrine [25]. Local release of neuroendocrine mediators coupled with specific receptor expression in immune cells establishes a functional neuroimmune connection capable of modulating various responses, including cytokine production. PE might be the result of increased pulmonary vascular permeability caused by the brain stem lesions and/or a systemic inflammatory response syndrome produced by the release of cytokines and chemokines. The clinical presentation of EV71 PE is caused by a hyperinflammatory syndrome resulting from hypercytokinemia and central nervous system inflammation of various inflammatory mediators. Some studies have shown that proinflammatory cytokines (interleukin (IL)-6, tumor necrosis factor (TNF)-*α*, and IL-1*β*) are associated with BE that is complicated by PE [17, 20]. A significant elevation of plasma IL-10, IL-13, and interferon (IFN)-*γ* levels is observed in patients with PE [19].

## 4. Cytokine in the Systemic Inflammatory Response of EV71 Infection

There is increasing evidence that proinflammatory and anti-inflammatory cytokines may play a central role in EV71 BE. Human P-selectin glycoprotein ligand-1 (PSGL-1; CD162) was identified as a receptor for EV71 in pathogenesis [26]. The interaction of EV71 with PSGL-1 on lymphocytes may induce production of the inflammatory cytokines involved in BE or PE [27].

Mononuclear phagocytic cells are the most important source of IL-6; however, IL-6 is also produced by T and B lymphocytes and numerous other cells [28]. Elevated plasma level of IL-6 was detected in EV71-infected patient with ANS dysregulation, the priming stage of PE [29]. The IL-1*β*, IL-6, and TNF-*α* levels in fatal patients with encephalitis plus PE were significantly higher than those of uncomplicated patients. Elevated IL-6 may represent the net effect of IL-1*β* and TNF-*α* biological actions. IL-6 > 70 pg/mL was found to be the best predictor of EV71 encephalitis with PE [17, 20].

EV71 infection significantly increased the release of IL-6 from dendritic cells [29]. IL-6 and T cells are shown to reduce mortality of EV71-infected mice by reducing tissue viral loads in a previous study [30]. However, Khong and coworkers reported that administration of anti-IL-6 neutralizing antibodies, at day 3 or 6 postinfection, after the onset of the clinical symptoms successfully improved the survival rates and clinical scores of the EV71-infected mice [31]. Compared to untreated infected controls, anti-IL-6-treated mice displayed reduced tissue damage, absence of splenic atrophy, and increased CD4+, CD8+ T cells, and B cells activation. Further, the anti-IL-6 antibody-mediated protection is independent of the virus load. However, anti-IL-6 treatment at the time of infection is detrimental to the mice. It means that IL-6 production is beneficial to the host early postinfection to trigger the antiviral host response through attraction of various immune cells; however, sustained high levels of IL-6 may cause tissue damage and immunopathology.

IL-10 is an important immunoregulatory cytokine with multiple biologic effects known to be produced by macrophages, dendritic cells, B cells, and various subsets of CD4+ and CD8+ T cells [32]. IL-10 can both impede pathogen clearance and ameliorate immunopathology. IL-10 was significantly higher in patients with PE than in those with ANS dysregulation or uncomplicated BE [19]. A close relationship exists between catecholamine and IL-10 release [33]. IL-10 can be modulated in several acute and chronic neuropathological conditions. This suggests that IL-10 plays a role in the immune-regulatory functions of the CNS. Thus, the systemic IL-10 increase in patients with PE appears to be triggered by persistent sympathetic activation as a consequence of direct brain stem destruction by the virus. IL-10 inhibits production of several proinflammatory mediators, including IL-1, IL-6, IL-8, granulocyte colony stimulating factor, and TNF-*α*, and upregulates the expression of the naturally occurring IL-1 receptor antagonist [32]. In an animal study of EV71 infection, anti-IL-6 treatment resulted in dramatically increased IL-10/IL-6 ratios, reflecting the upregulation of IL-10 production in the treated animals. Therefore, upregulation of IL-10 likely helped balance between proinflammatory and anti-inflammatory cytokines and subsequent tissue damage [31].

Interferons (IFN)-*γ*, a pleiotropic cytokine, is produced principally by CD4+ Th1 cells, cytotoxic CD8+ T cells, and NK cells. It is essential for both innate and adaptive immunity [34]. Elevated serum levels of IFN-*γ* in patients with uncomplicated BE and PE were found. Moreover, the kinetic analysis in patients with PE showed that the production of IFN-*γ* occurred 24 h after IL-10 production. Increased pulmonary vascular permeability may play a pivotal role in PE. IFN-*γ* can exhibit enhanced vascular permeability [35]. IFN-*γ*-mediated microvascular leakage occurs as a result of the reduced endothelial barrier and tight junction [36]. IL-10 is a cytokine synthesis inhibitor that will terminate the production of IFN-*γ*. IFN-*γ* production appeared later than that of IL-10 in PE patients, which suggests that IFN-*γ* might play an important role in the development of PE. Recently, IFN-*γ* was found to be significantly elevated in infected AG129 mice, which lack type I and II interferon receptors, that are susceptible to infection with a non-mouse-adapted EV71 strain via both the intraperitoneal and oral routes. The defect in IFN signaling may lead to some compensatory changes in the pattern of immune responses, which implies this model may not accurately reflect the immunopathogenesis seen in immunocompetent patients [37].

Macrophages, NK cells, dendritic cells, and fibroblasts were reported to produce IFN-I (*α* and *β*) in response to viral infection or exposure to microbial pathogens. The early induction and action of IFN-I result in cellular resistance to viral infection, inhibition of viral replication, and impediment of viral dissemination [38]. Liu and coworkers showed that polyriboinosinic: polyribocytidylic acid (poly(I : C)), a potent IFN inducer, improved the survival rate and decreased the tissue viral titers after EV71 challenge, which correlated with an increase in serum IFN-*α* concentration, the percentage of dendritic cells, the expression of major histocompatibility complex class II molecule and IFN-*α* in spleen of mice [39].Type I IFNs represent an essential innate defense mechanism for controlling EV71 infection in mice.

IL-13 is another cytokine produced by T cells that has potential anti-inflammatory activity and suppresses the cytotoxic functions of monocytes/macrophages [40]. Patients with PE were found to have higher IL-13 levels than those with uncomplicated BE [19]. High levels of IL-13, which are affected by endogenous IL-4 and required for airway hyperresponsiveness and mucus production, are found in patients with asthma and atopic dermatitis [41]. Plasma level of IL-4 was not changed in EV71-infected patients, but IL-13 levels were consistently elevated in all groups, uncomplicated BE, ANS dysregulation, and PE. Because IL-13 can act alone in the pulmonary model [42], overproduction of IL-13 might contribute to the pathogenesis of PE by increasing pulmonary vascular permeability. Further, exogenous treatment of IL-6, IL-13, and IFN-*γ* at day 3 postintracranial infection could induce mild PE and exacerbate pulmonary abnormality of EV71-infected mice. A synergistic proinflammatory cytokine response and damage to specific brain regions may be necessary for the development of EV71-induced PE [43]. Though several studies display the changes of cytokines and chemokines of EV71 infection, trigger the ANS dysregulation and elicit the PE were not showcased in animal model.

Chemokines, a group of small (8–12 kd) proteins, are key regulators of leukocyte migration and play important roles in many physiological and pathological immune and inflammatory contexts. Chemokines are characterized by the presence of 3 to 4 conserved cysteine residues and can be subdivided into 4 families based on the positioning of the N-terminal cysteine residues [44].

IL-8 was identified as a neutrophil-specific chemotactic factor and later classified as a member of the CXC chemokine family. The major effector functions of IL-8 are activation and recruitment of neutrophils to the site of infection or injury [45]. Patients with ANS dysregulation had higher plasma levels of IL-8 than patients with PE. IL-8 and other cytokines have been proposed to induce alterations in pulmonary permeability. IL-8 production contributes to the virus-induced effect on the cytoskeleton and tight junctions and thereby may modify transendothelial permeability [46]. This might account for the elevation of IL-8 in patients with ANS dysregulation and PE. In animal models of acute lung injury, neutralizing IL-8 reduced the severity of lung inflammation and tissue damage [47]. Treatment of the rabbits that showed extensive edema in the alveolar lumina with a humanized anti-IL-8 antibody prevented neutrophil infiltration in the lung in association with alleviated acute lung injury syndrome [48].

Because of the known increased expression of IFN-*γ* in Th1 diseases in children with EV71-associated BE, IFN-*γ*-induced protein-10 (CXCL10/IP-10), monokine induced by IFN-*γ* (CXCL9/MIG), and IFN-inducible T-cell *α* chemoattractant were studied. Plasma levels of IP-10, monocyte chemoattractant protein (MCP)-1, and MIG were significantly higher in patients with PE than in those with uncomplicated BE [22]. IP-10 is recognized as a biomarker that predicts severity of various diseases. IP-10 expression also can be upregulated by the Th1 cytokine IFN-*γ* during acute lung inflammation. This is consistent with previous findings that both circulating IFN-*γ* [19] and IP-10 levels were increased in patients with EV71 PE.

MCP-1 in the systemic inflammatory response has been suggested. The concentration of MCP-1 has been shown to increase in the plasma of patients with persistent acute respiratory distress syndrome [49]. This may provide an explanation for the increase in MCP levels in plasma when the disease progresses from uncomplicated BE to PE. MIG plays a role in host defense after viral infection [50]. Increased level of MIG in patients with EV71 PE supports the notion that MIG contributes to host defense by promoting a protective Th1 response. Overexpression of the chemokine cascade in the systemic compartment appears to play an important role in the elicitation of the immune response to EV71.

## 5. Cytokine in Central Nervous System of EV71 Infection

Cytokines are constitutively expressed in the CNS. Normally, the cellular expression of cytokines in the CNS is highly integrated and under tight regulatory control. However, in certain pathological conditions, cytokine production may become spatially and temporally dysregulated, leading to inappropriate production. Although the blood-brain barrier (BBB) is relatively impermeable to cytokines owing to their size and hydrophilicity, in EV71 BE, the integrity of the BBB may be compromised permitting cytokine action within the CNS. In the inflammatory process of echovirus 30 meningitis, cytokine network shifts from production of proinflammatory cytokines (IL-6, IL-8, and IFN-*γ*) to that of anti-inflammatory cytokines (IL-10 and TGF-*β*1) during or after the period when the virus is eliminated from the cerebrospinal cavity [51].

Lin and coworkers examined the relationship between level of IL-6 in the cerebrospinal fluid (CSF) and the CNS involvement of EV71 infection [20]. The median CSF level of IL-6 was significantly higher during the first or second day of CNS involvement. However, CSF levels of IL-6 were not significantly different among patients with different clinical EV71 syndromes, PE, encephalitis and/or poliomyelitis-like syndrome, and aseptic meningitis during the acute stage of CNS involvement [20]. However, another study demonstrated the mean CSF concentrations of IL-6 were elevated significantly in children with PE, and ANS dysregulation as compared to children with uncomplicated BE [21]. The CSF concentrations of IL-6 were elevated by the diseases severity of EV71 infection. The source of IL-6 appears to be the brain, since CSF levels of IL-6 exceed those in plasma. This suggests that IL-6 may contribute to the overwhelming disease process.

Children with EV71 PE or ANS dysregulation had higher IFN-*γ* levels in CSF than those with isolated BE and echovirus meningitis. This suggests that IFN-*γ* is responding maximally to severe infection [21]. IFN-*γ* is not normally present in the brain parenchyma. IFN-*γ* appears to promote gliosis and inflammation by its effect on astrocytes. Inflammation protects the brain from infection, but it aggravates injury. This suggests a detrimental effect of IFN-*γ* on the CNS [52].

IL-1 activates microglia and vascular endothelial cells to recruit peripheral leukocytes and produce neuroinflammation. Elevated IL-1*β* levels were found only in the CSF and not in the plasma of EV71-infected patients with PE [21]. Hosoi et al. [53] found increased levels of IL-1*β* mRNA in the brain in the absence of an increase in circulating IL-1*β* in rats. These findings support the notion that IL-1*β* is probably synthesized in the CNS in response to severe EV71 infection. IL-1 can also regulate neurotransmission mediated by amines such as norepinephrine and dopamine. The activity of the locus coeruleus, which is the most important source of norepinephrine inside the brain, was increased after in vivo IL-1*β* microinjection in this area; this effect was blocked by IL-1*β* receptor antagonist [54]. This may provide explanation of elevated IL-1*β* level in CSF of PE patients, following the stage of ANS dysregulation.

IP-10 and its receptor, CXCR3, are expressed by the CNS and by CNS infiltrating lymphocytes, respectively, only in patients with ongoing CNS inflammation, suggesting an important role for these molecules in the pathogenic process. Increased IP-10 concentration in CSF in patients with enteroviral meningitis was observed [55]. CSF levels of IP-10 and IL-8 in patients with EV71 BE were significantly higher than the plasma levels in the control subjects [22]. IP-10 is prominently expressed within the CNS of mice after infection with viral encephalitis [56]. Early expression of IP-10 within the CNS after virus infection is important in initiating and maintaining a protective Th1 immune response. This is characterized by high-level production of the antiviral cytokine IFN-*γ* [56, 57]. Early expression of IP-10 appears to be beneficial by attracting Th1 T lymphocytes into the CNS, which participate in viral clearance. Further study is needed to determine how IP-10 neutralizing agents or CXCR3 receptor antagonists might be applied to treating human disease.

CSF levels of MIG were observed to be significantly more elevated in patients with EV71 PE than in those with EV71 ANS dysregulation and uncomplicated BE. The CSF to plasma ratio for MIG tended to increase with increasing severity of disease [22]. In a study of murine brain endothelial cells, MIG was induced following treatment with a cytokine cocktail containing IFN-*γ*, TNF-*α*, and IL-1*β* [58]. The coordinate regulation of IP-10 and MIG was mediated by IFN-*γ* in cultured murine astrocytes and microglia [59]. This may support the previous findings, the increased CSF level of MIG may relate to the increased CSF level of IL-1*β* and IFN-*γ* in patients with PE.

## 6. Modulation of Systemic Inflammatory Response of EV71 Infection

Intravenous immune globulin (IVIG) is a polyclonal immunoglobulin derived from large pools of human serum. IVIG has been investigated as a therapeutic modality for many viral infectious conditions. The plausible mechanisms of action of IVIG that have been reported to cause an amelioration of inflammatory processes include interaction with Fc receptors, induction of apoptosis, blockade of costimulatory molecules, interference with the cytokine network, and neutralization of pathogenic antibodies [60].

IVIG has been used prophylactically and therapeutically against neonatal enterovirus infections and in immunocompromised hosts [61]. IVIG injection decreases plasma catecholamines in coxsackievirus B3 myocarditis, suggesting that immunoglobulin exerts its cardioprotective effect through sympathetic modulating actions [62]. There is considerable evidence linking cytokine-mediated severe systemic inflammatory responses to PE and the other adverse outcomes in patients with EV71-associated BE [17, 19, 21]. Modulating cytokine expression by IVIG may offer a strategy for clinical practice. A previous study demonstrated a decrease in the plasma concentration of IL-6, IL-8, IL-10, IL-13, and IFN-*γ* following administration of IVIG in patient with ANS dysregulation and PE [29]. These changes may be responsible for the rapid improvement in symptoms in some treated patients. Patient with ANS dysregulation is the critical timing to receive IVIG infusion. It is possible that a more favorable survival might have been obtained by modulating cytokine storm and reducing sympathetic activity.

Milrinone, a bipyridine phosphodiesterase (PDE) III inhibitor, is a member of both inotropic and vasodilatation characters. Milrinone increases cardiac output and reduces systemic vascular resistance and pulmonary capillary wedge pressure without excessive increases in myocardial oxygen consumption [63]. Inhibition of cyclic adenosine 3′,5′-monophosphate (cAMP) degradation by intracellular PDE3 may attenuate inflammation, reduce edema formation, improve endothelial function, and induce pulmonary vasodilation [64]. A study was designed to evaluate the potential therapeutic effect of milrinone in the treatment of patients with EV71-induced PE [65]. The mortality was lower in the milrinone-treated than nontreated group. Sympathetic tachycardia, white blood cell and platelet counts were decreased. There was a significant decrease in plasma level of IL-13 in milrinone-treated patients compared to controls. Milrinone therapy may provide a useful therapeutic approach for treating life-threatening EV71 infections.

There are substantial evidences that blood purification may play a potential role in the treatment of patients with hypercytokinemia. Mechanisms of cytokine removal by blood purification were proposed include convection, diffusion, and adsorption. The extent of cytokine removal achieved by a blood purification therapy utilizing any of these mechanisms alone or in combination depends mainly on the material and shape of the hemofilter/hemodialyzer/adsorber used as well as the operating conditions of the blood purification system [66]. Studies have led to the idea that adsorption contributes more strongly to cytokine removal than convection or diffusion in some membrane materials [67]. Whereas the appropriate timing and duration of intervention and the possible clinical effectiveness are important and should be take into consideration in severe EV71-infected patients.

## 7. Conclusions

**Figure 1:**
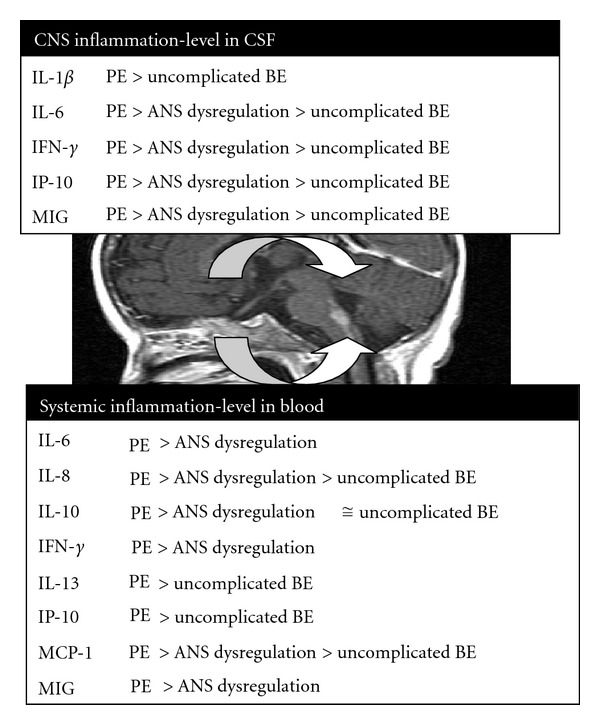
Inflammatory responses in cerebrospinal fluid and plasma of patients with enterovirus 71 brain stem encephalitis by disease severity.

The production of inflammatory cytokines and chemokines is a unique aspect of the immune responses in the CNS and systemic compartment to EV71 infection (Figure 1). Cytokines and chemokines released by EV71 infected immune cells may contribute directly or indirectly to the disease severity. The use of IVIG and milrinone represents an appropriate approach to the treatment of the inflammatory responses elicited by severe EV71 infection. Alternative modalities for controlling the cytokine network have been explored experimentally. A better understanding of the fundamental mechanisms and the engaged inflammatory signal-transduction pathways of the cytokines production will be expected to be of value in the treatment of inflammation and EV71 infection.
